# High cGAS-STING expression associates with improved efficacy of neoadjuvant chemo-immunotherapy in head and neck squamous cell carcinoma

**DOI:** 10.3389/fonc.2025.1584061

**Published:** 2025-07-15

**Authors:** Miao Wang, Menglin Shi, Yiming Ding, Zishanbai Zhang, Yuze Ge, Zhixin Li, Yixin Jing, Honglian Hu, Xiaohong Chen

**Affiliations:** ^1^ Department of Otolaryngology-Head and Neck Surgery, Beijing Tongren Hospital, Capital Medical University, Beijing, China; ^2^ Department of Biochemistry and Molecular Biology, Capital Medical University, Beijing, China; ^3^ Department of Oncology Center, Beijing Tongren Hospital, Capital Medical University, Beijing, China

**Keywords:** head and neck squamous cell carcinoma, neoadjuvant chemoimmunotherapy, cGAS-STING, predictive markers, T cells

## Abstract

**Purpose:**

Neoadjuvant chemo-immunotherapy (NACI) has demonstrated significant clinical advantages in head and neck squamous cell carcinomas (HNSCC), while clinical responses vary in different patients. This study investigated the correlation between the cyclic GMP-AMP synthase (cGAS, *CGAS*) and the stimulator of interferon genes (STING, *STING1*) expressions and the efficacy of NACI in HNSCC.

**Methods:**

The correlation between *CGAS* and *STING1* expressions and chemotherapy/immunotherapy drug sensitivity was analyzed using the GDSC and TCIA dataset. The study enrolled 38 HNSCC patients receiving NACI, with protein expressions of cGAS and STING evaluated via immunohistochemistry. The T cell abundance and tumor-T cell interactions in different *CGAS* and *STING1* expression groups were analyzed using bulk RNA-seq and scRNA-seq data from open databases.

**Results:**

The mRNA expressions of *CGAS* and *STING1* were negatively correlated with the IC50 of docetaxel and positively correlated with the efficacy of anti-PD-1 treatment (p<0.05). In the real-world cohort, cGAS and STING expressions were both positively related to NACI efficacy (p<0.05). The mRNA expressions of *CGAS* and *STING1* were positively correlated with the abundance of Act-CD4 (*CGAS*: rho=0.416, p<2.21e-16; *STING1*: rho=0.26, p=1.82e-09), Act-CD8 (*CGAS*: rho=0.089, p=0.0425; *STING1*: rho=0.303, p=1.98e-12), NKT cell (*CGAS*: rho=0.255, p=0.3.78e-09; *STING1*: rho=0.375, p=2.2e-6). Tumor cells with increased expression of *CGAS* or *STING1* showed enhanced interactions with T cells.

**Conclusion:**

This study confirms the positive correlation between cGAS and STING expressions and NACI efficacy, suggesting their role in immune activation and potential as biomarkers for predicting NACI efficacy in HNSCC.

## 1 Introduction

Head and neck cancer ranks as the eighth most prevalent malignancy globally, with an estimated 890,000 new cases and 450,000 deaths annually in 2022. Among these, head and neck squamous cell carcinomas (HNSCC) are the most common type (1). The combination of surgery, chemotherapy, and radiation therapy maximizes treatment efficacy, yet the 5-year overall survival (OS) remains only 50%. Once the disease has recurred, the 1-year OS rate is approximately 15%, with a median OS of 10 to 14 months (2). In recent years, the rise of immunotherapy has significantly improved patient outcomes. KEYNOTE 040, CheckMate 141, and KEYNOTE 048 trials have shown that immunotherapy demonstrates encouraging efficacy in metastatic/recurrent (R/M) HNSCC, with response rates ranging from 15% to 23% (3–5). Previous preclinical studies suggest that immunotherapy is more effective in the neoadjuvant setting than the adjuvant setting (6). Multiple trials on NACI can achieve a pathological complete response rate of approximately 33.3% to 55.6% in HNSCC patients (7–9). Therefore, identifying predictive biomarkers for therapeutic efficacy to select patient subgroups amenable to precision treatments has become a critical priority in NACI.

The stimulator of interferon genes (STING, *STING1*) pathway, activated by the cyclic GMP-AMP synthase (cGAS, *CGAS*) detection of aberrant double-stranded DNA (dsDNA), leading to a type-I interferon (IFN) response (10). The most effective regimen for neoadjuvant treatment of locally advanced HNSCC is confirmed as chemotherapy combined with immunotherapy (11). Chemotherapeutic agents, such as cisplatin, can activate the STING pathway, enhance cytotoxic T cell infiltration and increasing sensitivity to immunotherapy (12, 13). However, STING knockout has been demonstrated to significantly enhance resistance to cisplatin in HNSCC (14). Moreover, deletion of cGAS and STING reverses the anti-tumor effects of chemo-immunotherapy in small cell lung cancer (15). These findings suggest that the expression levels of cGAS and STING may serve as predictive biomarkers for response to chemo-immunotherapy.

This study evaluates the association between cGAS-STING expression and NACI efficacy in HNSCC. It also explores the immunological function of cGAS-STING and its potential as a predictive biomarker for therapeutic response.

## 2 Materials and methods

### 2.1 Open-database sources

*CGAS* and *STING1* expression levels in HNSCC from The Cancer Genome Atlas database (TCGA, http://portal.gdc.cancer.gov/) were analyzed. Regarding the high/low grouping, we dichotomized samples into high-expression and low-expression groups using the median value. The correlation between CGAS and STING expression and IC50 values for common chemotherapeutic drugs (cisplatin, docetaxel, and 5-Fluorouracil) was analyzed using the Genomics of Drug Sensitivity in Cancer (GDSC, http://www.cancerrxgene.org/) and processed with the R package ‘oncoPredict. Additionally, immunotherapy data from The Cancer Immunome Atlas (TCIA, http://tcia.at/) was analyzed to assess the effectiveness of immunotherapy between the cGAS-STING high and low expression groups within the TCGA-HNSC cohort. The correlation between cGAS and STING expression and the abundance of immune cells was analyzed by TISIDB (http://cis.hku.hk/TISIDB/). Tumor-T cell interactions were explored using scRNA-seq data from the GEO database.

### 2.2 Clinical patients

This study enrolled HNSCC patients who received NACI at Beijing Tongren Hospital, Capital Medical University. Between June 2019 and April 2024, 38 patients received NACI. Eligibility criteria were as follows: (1) age ≥18 years, (2) pathologically confirmed squamous cell carcinoma, (3) no prior treatments before neoadjuvant therapy, and (4) at least one measurable or evaluable lesion according to Response Evaluation Criteria in Solid Tumors 1.1 (RECIST 1.1) (16).

### 2.3 Data collection

Clinicopathological characteristics, including age, gender, anatomical subsite, TNM staging (according to the 8th edition of the American Joint Committee on Cancer Staging Manual), histological classification (poorly, moderately, well differentiated), smoking history, and alcohol consumption, were retrieved from the medical records system at Beijing Tongren Hospital, Capital Medical University.

### 2.4 Treatment regimes

The NACI group received pembrolizumab or tislelizumab in combination with the TP, PF, or TPF regimens. The TP regimen comprised paclitaxel 135 mg/m² and cisplatin 100 mg/m² on day 1, while the TPF regimen incorporated 5-fluorouracil 1000 mg/m² from days 1 to 5. The PF regimen consisted of cisplatin 100 mg/m² on day 1 and 5-fluorouracil 1000 mg/m² from days 1 to 5.

### 2.5 Efficacy assessment

The efficacy of NACI was evaluated based on the clinical and pathological responses. Clinical responses were evaluated based on radiologic evaluation of tumor size by magnetic resonance imaging (MRI) before and after neoadjuvant therapy according to RECIST, version 1.1 (16). Patients were classified as responders (complete response (CR) plus partial response (PR)) or non-responders (stable disease (SD)), and progressive disease (PD). Pathological responses were evaluated based on the percentage of residual viable tumor (RVT). We classified patients into major pathologic response (MPR) (defined as ≤10% RVT in the resected tumor specimen, including pathological complete response (pCR) (no RVT)), or incomplete pathologic response (IPR; defined as >10% RVT in the resected tumor specimens.

### 2.6 IHC analysis

Fixed tissue samples were deparaffinized with xylene and graded ethanol, followed by antigen retrieval using EDTA (pH 9.0) or sodium citrate buffer (pH 6.0). Samples were then treated with 30% hydrogen peroxide for 10 minutes at room temperature, blocked with goat serum for 1 hour at 37°C, and incubated overnight at 4°C with primary antibodies: cGAS (Cell Signaling Technology, 79978S, 1:100), STING (ProteinTech, 19851-1-AP, 1:2000). Horseradish peroxidase activity was detected using a PV two-step IHC kit. Negative controls used rabbit or mouse IgG. Staining intensity was analyzed using ImageJ software, and images were captured using a Leica microscope.

### 2.7 Statistical analysis

Data are presented as mean ± SD or mean ± SEM unless otherwise stated. To assess the predictive performance of cGAS and STING, receiver operating characteristic (ROC) curves were constructed, and the areas under the curves (AUCs) were calculated. ROC curves were plotted using the ‘pROC’ package. Group comparisons were performed using a two-tailed unpaired Student’s t-test. A p-value of less than 0.05 was considered statistically significant. All analyses were conducted using GraphPad Prism 8.3.0 or R 4.2.3 software.

## 3 Results

### 3.1 cGAS-STING expressions were positively related to chemotherapy and immunotherapy efficacy in TCGA-HNSC cohort

**Figure 1:**
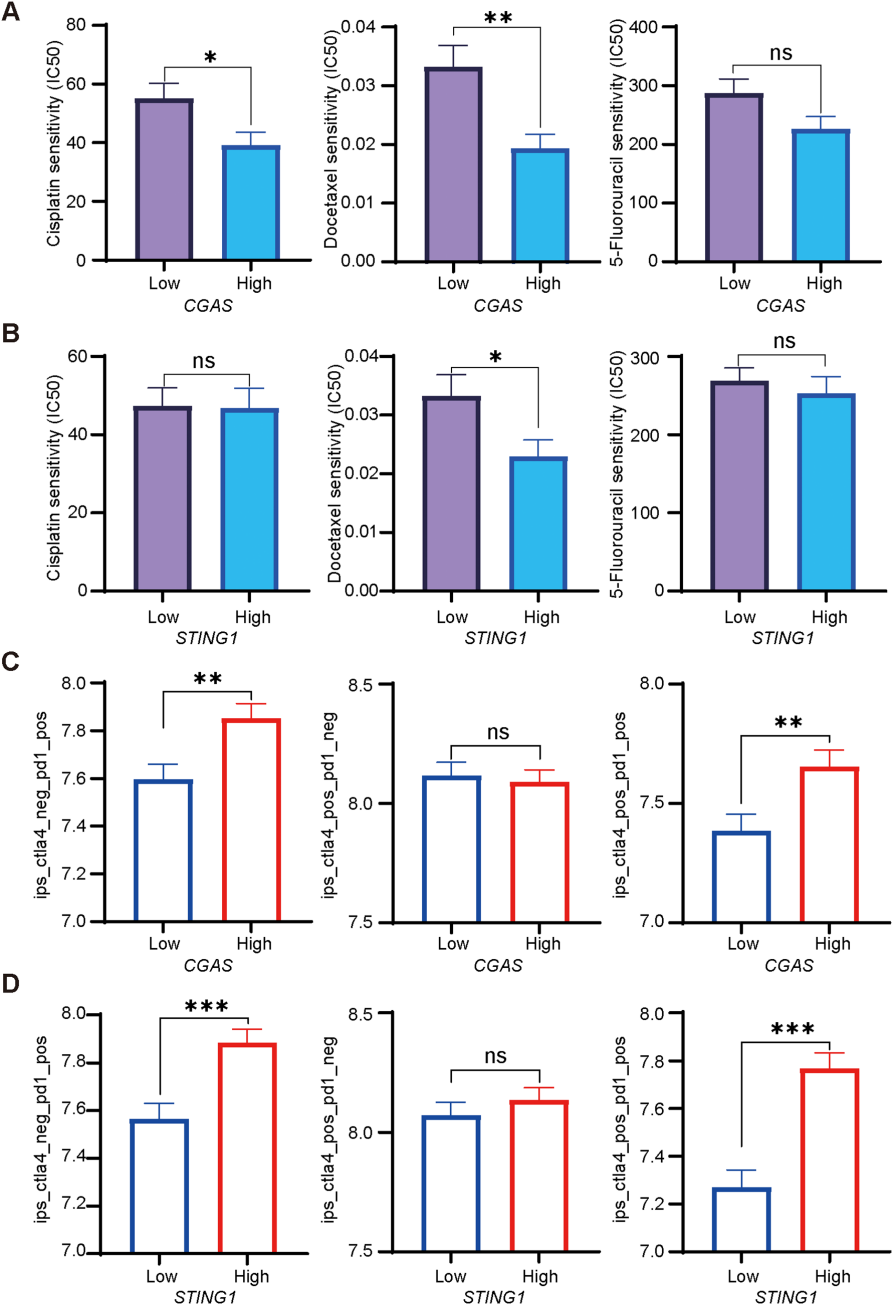
Chemotherapeutic and immunotherapy responses based on cGAS-STING expression in HNSCC. **(A, B)**. Relationships between high and low *CGAS* or *STING1* expression groups and IC50 of cisplatin, docetaxel, and 5-fluorouracil. **(C, D)**. The IPS assessment of anti-PD-1, anti-CTLA-4, and their combination therapies, analyzing the differences between patients with high and low *CGAS* and *STING1* expression groups. PD-1, programmed cell death protein 1; CTLA-4, cytotoxic T lymphocyte antigen 4. *p<0.05, **p<0.01, ***p<0.001.

Sensitivity differences of cisplatin, docetaxel, and 5-fluorouracil in the TCGA-HNSC cohort was conducted. Higher IC50 values indicate worse chemotherapy efficacy. The results showed that the *CGAS* high-expression group had lower IC50 values of cisplatin and docetaxel, suggesting increased sensitivity to these chemotherapy treatments (**Figure 1A**). No significant difference in 5-fluorouracil treatment response was observed between high- and low-expression groups (**Figure 1A**). Additionally, elevated *STING1* expression was related to higher docetaxel sensitivity, and was not associated with the efficacy of cisplatin and 5-fluorouracil (**Figure 1B**).

To assess the predictive value of cGAS-STING pathway expression for immunotherapy, we evaluated the IPS scores for anti-programmed cell death protein 1 (PD-1) and anti-cytotoxic T lymphocyte antigen 4 (CTLA-4) therapies. Higher scores indicate better outcomes. Individuals with elevated *CGAS* expression showed higher IPS scores for anti-PD-1 monotherapy alone or combined with anti-CTLA-4 treatment, suggesting a stronger response (**Figure 1C**). High *STING1* expression was associated with increased IPS for anti-PD-1 alone or combined with anti-CTLA-4 treatment (**Figure 1D**).

### 3.2 cGAS and STING expression levels were positively correlated with NACI response in HNSCC patients

Based on our analysis suggesting that cGAS and STING may influence chemotherapy and immunotherapy efficacy, we established a retrospective cohort of HNSCC patients treated with NACI to further investigate our findings. Baseline characteristics are presented in **Table 1**. A total of 38 patients were enrolled and received NACI therapy, 31 of whom proceeded to surgical resection, while 7 received non-surgical therapy. Among the 19 responders, 10 achieved pathological complete response (pCR). Over 75% had a history of smoking or alcohol use. The most common primary tumor sites were the hypopharynx (50.00%), followed by oropharynx (23.68%), larynx (21.05%), and nasal cavity and sinus (5.26%). Histological classification included poorly differentiated (44.74%), moderately differentiated (31.58%), and well differentiated (7.89%). A significant majority (86.84%) had advanced disease (stages III and IV).

**Table 1:** Baseline characteristics of patients treated with neoadjuvant chemo-immunotherapy.

Patient characteristics	N	%
Age		
≤60	23	60.53
>60	15	39.47
Gender		
Male	37	97.37
Female	1	2.63
Alcohol consumption		
No	4	10.53
Yes	34	89.47
Smoking history		
No	8	21.05
Yes	30	78.95
Tumor site		
Nasal Cavity and Sinus	2	5.26
Oropharynx	9	23.68
Hypopharynx	19	50.00
Larynx	8	21.05
Histological classification		
Poorly differentiated	17	44.74
Moderately differentiated	12	31.58
Well differentiated	3	7.89
HPV status		
negative	24	63.16
positive	7	18.42
T stage		
1	3	7.89
2	12	31.58
3	11	28.95
4	12	31.58
N stage		
0	9	23.68
1	10	26.32
2	19	50.00
Clinical stage		
I	4	10.53
II	1	2.63
III	8	21.05
IV	25	65.79
Radiographic response		
Responders	19	50.00
Non-responders	19	50.00
Pathological response		
pCR	13	34.21
MPR	9	23.68
IPR	9	23.68
NA	7	18.42

**Figure 2:**
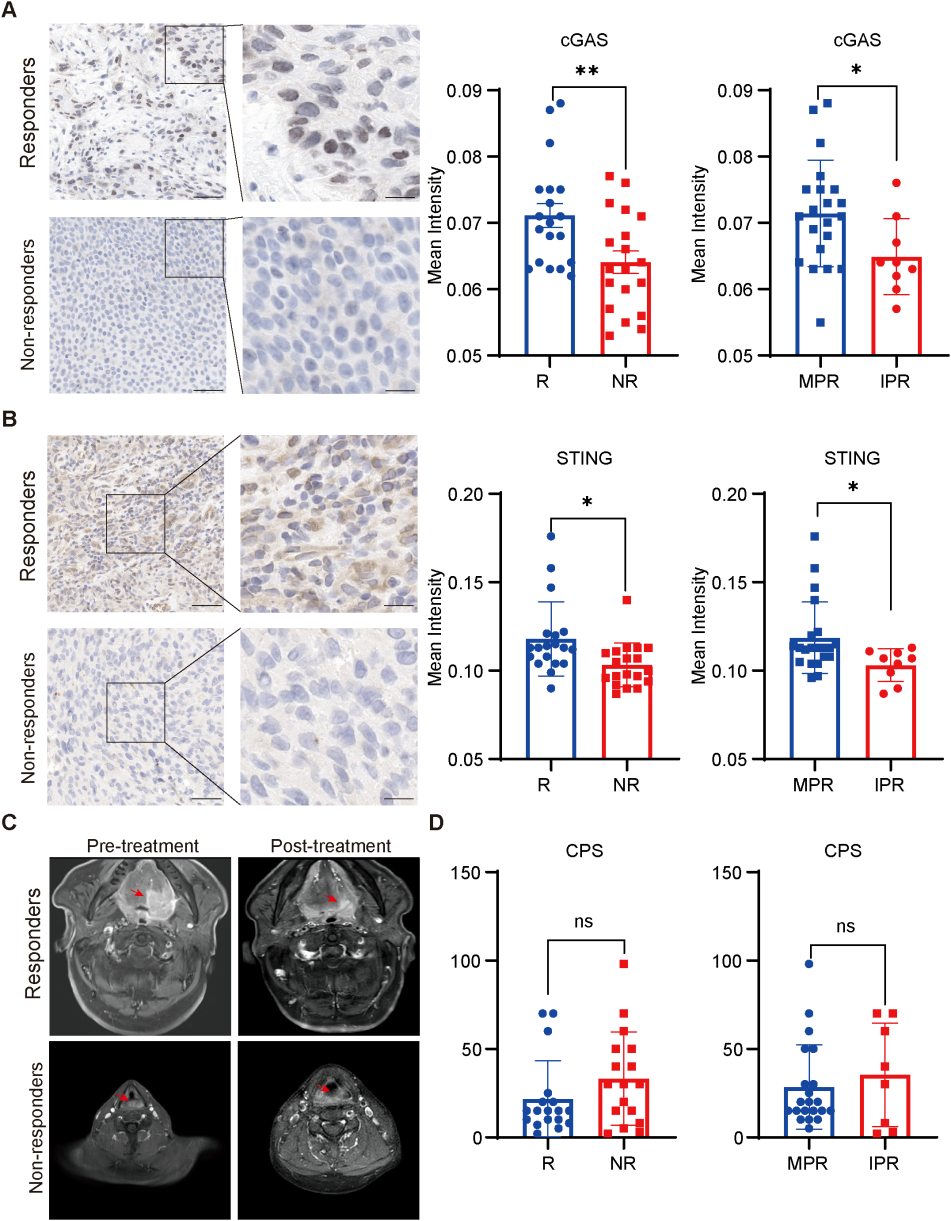
Correlation between cGAS-STING pathway levels and the response to NACI **(A, B)**. Immunohistochemical staining for cGAS and STING in the responders (R; n=19) and non-responders (NR; n=19) patients to NACI. expression of cGAS and STING in tumors was assessed in the MPR group (n = 22) and IPR group (n = 9). Scale bar: left panel, 50um; right panel, 25um. (C). Representative images showing the tumor size in responders and non-responders before and after NACI treatment, respectively. (D). CPS scores were assessed in the responders (R; n = 19) and non-responders (NR; n = 19) with NACI. CPS was assessed in the MPR group (n = 22) and IPR group (n = 9). MPR: major pathologic response; IPR: incomplete pathologic response. *p<0.05, **p<0.01.

Tumor samples were collected from the patients before receiving NACI treatment, and expression levels of cGAS and STING in tumor cells were evaluated by performing immunohistochemistry. The protein levels of cGAS and STING were not associated with clinical characteristics including HPV status (**Supplementary Table S1**). Notably, we found that cGAS and STING expression levels were significantly higher in responders than in non-responders (**Figures 2A–C**). To better assess the contribution of cGAS and STING to the efficacy of NACI, we evaluated the pathological responses based on the percentage of RVT cells. As anticipated, the expression of cGAS or STING had a higher level in the patients with MPR than in those with IPR (**Figures 2A, B**). Additionally, clinical characteristics of these patients has no relationship with the response to NACI (**Supplementary Table S2**). To date, the CPS score has been developed to predict the response to anti-PD-1 therapy in cancer patients (4), while our results demonstrated the expression levels of PD-L1 CPS expression did not significantly correlate (**Figure 2D**).

**Figure 3:**
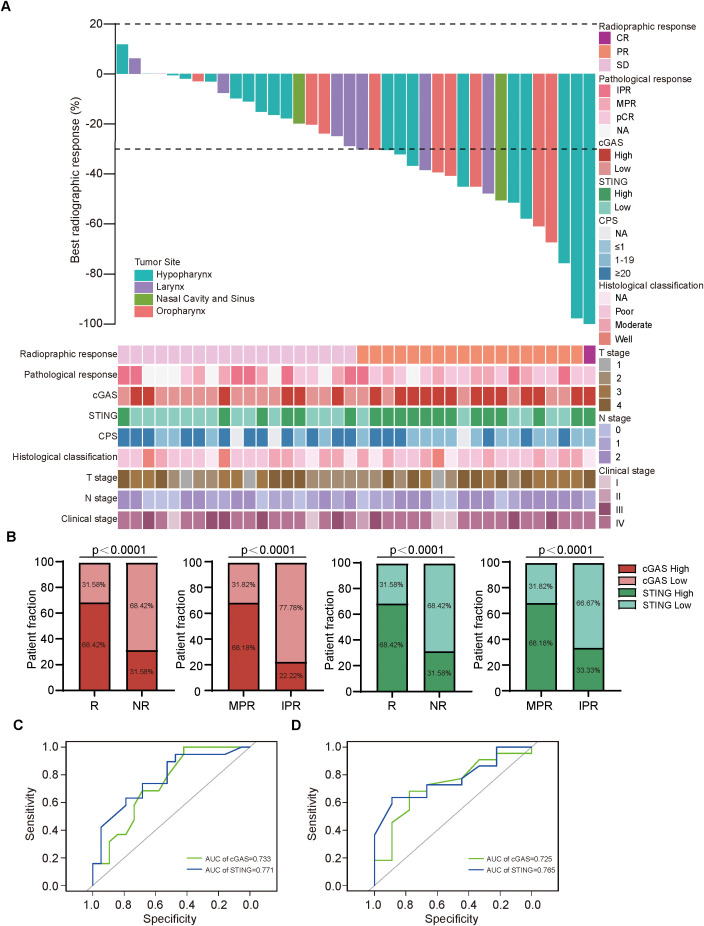
cGAS and STING expressions were positively related to responses to NACI in HNSCC patients **(A)**. Waterfall plots depicting the maximum percentage change in target lesion size during neoadjuvant chemo-immunotherapy of HNSCC patients (n = 38). **(B)** Distribution of cGAS and STING expression between responders (R; n=19) and non-responders (NR; n=19), patients with MPR (n = 22) and IPR (n = 9). **(C)** Predictive value of cGAS and STING in discriminating responders and non-responders via ROC analysis. **(D)** Predictive value of cGAS and STING in discriminating MPR and IPR groups via ROC analysis. MPR, major pathologic response; IPR, incomplete pathologic response.

To evaluate the correlation between the expression of cGAS and STING and various clinicopathological factors in our cohort, the waterfall plot of radiological responses for individual patients is shown in **Figure 3A**. cGAS and STING expressions showed no correlation with common clinical characteristics, including age, gender, alcohol consumption, and smoking history (**Supplementary Table S1**). Our results indicated that 68.42% (13/19) of responders exhibited high cGAS expression, compared to 31.58% (6/19) of non-responders, suggesting that elevated cGAS expression is associated with a positive therapeutic response. Similarly, 68.18% (15/22) of patients in the MPR group had high cGAS expression, whereas only 22.22% (2/9) of patients in the IPR group displayed high expression, further supporting its correlation with favorable treatment outcomes. Likewise, 68.42% (13/19) of responders displayed high STING expression, compared to 31.58% (6/19) of non-responders, indicating that higher STING expression correlates with a better response to treatment. A similar trend was observed in the MPR group, where 68.18% (15/22) exhibited high STING expression, compared to 33.33% (3/9) in the IPR group (**Figure 3B**). Next, we assessed the predictive value of cGAS and STING in distinguishing responders from non-responders and MPR from IPR groups. Notably, both cGAS and STING expression levels could effectively differentiate potential NACI responders from non-responders (AUC = 0.733 for cGAS and AUC = 0.771 for STING) (**Figure 3C**), as well as MPR from IPR groups (AUC = 0.725 for cGAS and AUC = 0.765 for STING) (**Figure 3D**).

### 3.3 Tumor cells with higher cGAS and STING expression exhibited stronger receptor-ligand interactions with T cells

**Figure 4:**
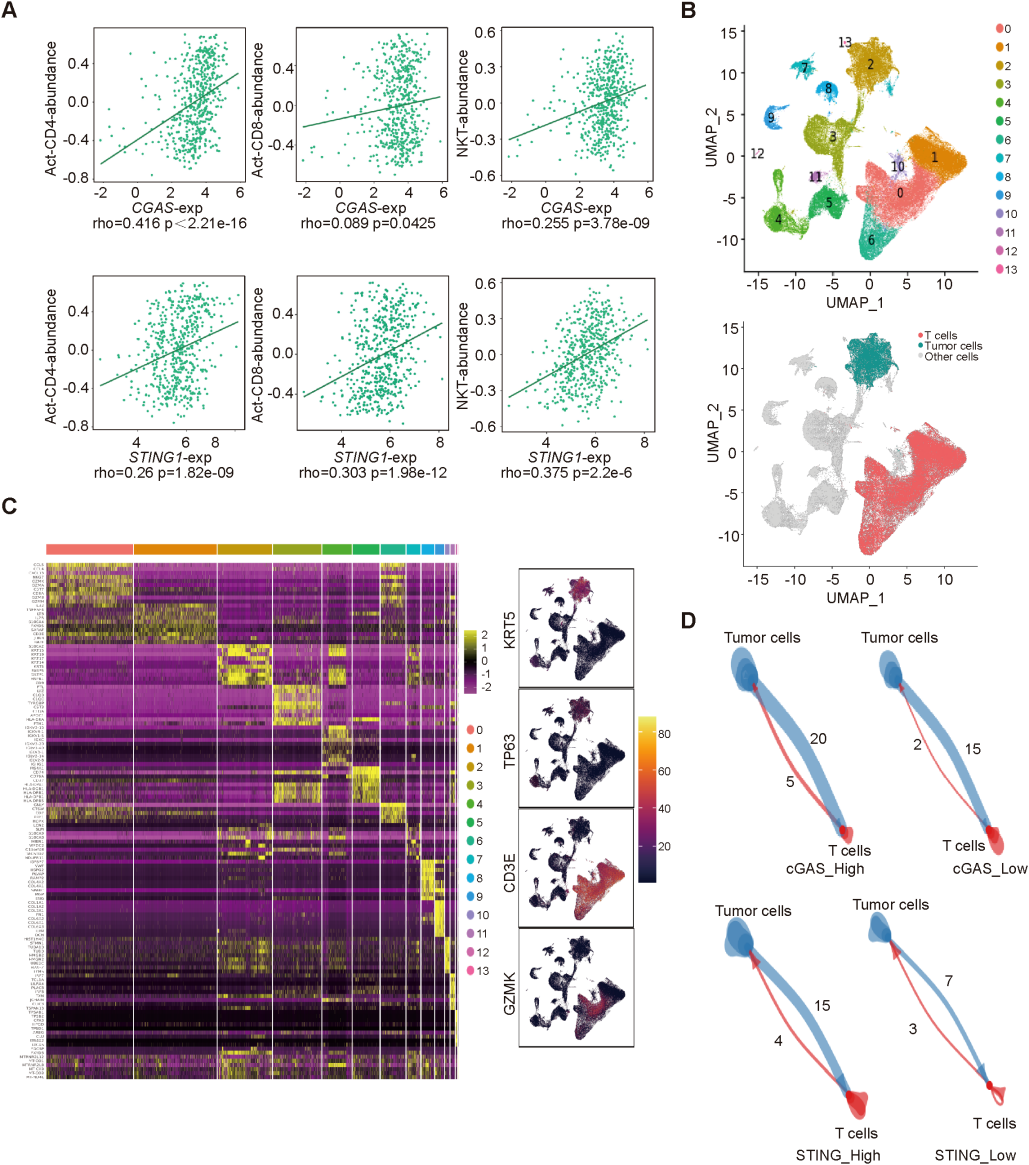
The correlation between cGAS/STING expressions and immune cells **(A)**. the relationship between cGAS/STING expressions and Act-CD4, Act-CD8, NKT cell abundance. **(B)** Uniform manifold approximation and projection (UMAP) visualization of single-cell transcriptomic profiles combined from all 24 samples. **(C)** Identification of genes that are dominantly expressed in each major cell type. **(D)** The cell-cell interactions between high- and low- cGAS/STING tumor cells and T cells.

To further investigate the functional characterization of cGAS and STING in the tumor microenvironment, we utilized the TISIDB database to explore the correlation between *CGAS* and *STING1* expressions and immune cell abundances. Both cGAS and STING1 expressions were positively correlated with Act-CD4 (*CGAS*: rho=0.416, p<2.21e-16; *STING1*: rho=0.26, p=1.82e-09), Act-CD8 (*CGAS*: rho=0.089, p=0.0425; *STING1*: rho=0.303, p=1.98e-12), NKT cell (*CGAS*: rho=0.255, p=0.3.78e-09; *STING1*: rho=0.375, p=2.2e-6) abundance, which are critical for immunotherapy (**Figure 4A**). Additionally, we performed scRNA-seq data analysis in primary tumor tissues of HNSC-GSE234933. Using uniform manifold approximation and projection (UMAP), we identified 14 cellular clusters, including tumor and T cells (**Figures 4B, C**). We then a cell-cell interaction analysis was conducted to further investigate the predictive value of cGAS and STING. Based on the expression levels of *CGAS* or *STING1* in tumor cell clusters, the samples were divided into high- and low-expression groups. We found that tumor cell clusters with higher *CGAS* or *STING1* expression exhibited stronger receptor-ligand interactions with T cells (**Figure 4D**). These findings suggest that cGAS and STING are strongly associated with T cell activity and enhance the response to NACI in HNSCC.

## 4 Discussion

Immune checkpoint inhibitors have emerged as a promising approach for the treatment of HNSCC. The KEYNOTE-048 trial demonstrated that pembrolizumab, when combined with chemotherapy, improved overall survival (OS) compared to the EXTREME regimen in patients with recurrent or metastatic (R/M) HNSCC, with an OS of 13.0 months versus 10.7 months (HR 0.77 [95% CI 0.63–0.93], p=0.0034) in the overall population (3, 4). Furthermore, several studies have reported that NACI can achieve pCR rates exceeding 50% in HNSCC patients (7, 8). However, patients who exhibit poor responses to NACI may experience treatment-related complications, potentially delaying radical surgery or concurrent chemoradiotherapy, without resulting in any improvement in prognosis (17). Therefore, identifying clinical biomarkers that can accurately predict the therapeutic response to NACI in HNSCC is of significant clinical importance. In this study, HNSCC patients with elevated levels of cGAS and STING demonstrated a higher response rate to NACI, as evidenced by both open-access database and real-world data.

Currently, CPS is the primary biomarker for predicting tumor immunotherapy efficacy, with higher CPS values associated with better responses to PD-1 therapy (4). In the KEYNOTE-012 study, the response rate was 21% in PD-L1+ patients compared to 6% in PD-L1− patients, as assessed by CPS (18). However, clinical trial data on its predictive value are inconsistent, and the long-term analysis of CheckMate-141 indicated that PD-L1− patients may also benefit from immunotherapy treatment (19, 20). Moreover, the CPS score did not correlate with NACI efficacy in the present cohort, with a substantial proportion of non-responders exhibiting CPS ≥20. This undermines the predictive accuracy of PD-L1 expression and highlights the limited predictive value of CPS. Immunotherapy efficacy is largely influenced by tumor antigen levels and inflammation, making these factors essential for optimizing treatment strategies (21, 22). Recent studies demonstrated that activation of the cGAS-STING pathway enhances antigen presentation by dendritic cells and stimulates the secretion of chemokines, such as CXCL9 and CXCL10, which recruit CD8+ T cells and NK cells to the tumor microenvironment, thereby enhancing the efficacy of immunotherapy (23–26). In this study, we assessed the mRNA expression of *cGAS* and *STING1* and found a positive correlation between their expression levels and the efficacy of chemotherapy and immunotherapy. Furthermore, the protein levels of cGAS and STING were significantly higher in responders to NACI treatment compared to non-responders. Additionally, patients with MPR had higher protein levels of cGAS and STING than those with IPR.

The tumor microenvironment plays a pivotal role in malignancy progression and significantly impacts the response to immunotherapy (27). Three primary immune profiles are associated with immunotherapy response: immune‐inflamed phenotype, the immune‐excluded phenotype and the immune‐desert phenotype (28). Notably, the immune-inflamed phenotype, characterized by abundant immune cell infiltration, generally correlates with better responses to immunotherapy in cancer patients (29, 30). In our study, we observed that the mRNA expressions of *CGAS* and *STING1* were positively correlated with the abundance of activated CD4+ T cells, activated CD8+ T cells, and NKT cells. Furthermore, elevated expression levels of CGAS and STING1 in tumor cells promoted their interaction with T cells, which is crucial for enhancing the efficacy of immunotherapy.

In conclusion, we conducted a systematic and comprehensive analysis of the role of cGAS-STING pathway expression in predicting the efficacy of NACI in HNSCC. However, several limitations should be noted. First, our study is a single-center retrospective analysis, and the limited sample size may affect the statistical power of subgroup analyses, requiring validation in larger cohorts. Second, although the correlation analysis indicates that the cGAS-STING pathway may enhance therapeutic efficacy through the modulation of T cell infiltration, further studies involving gene knockout or overexpression models are required to establish a causal relationship.

## 5 Conclusion

In summary, our study demonstrated that cGAS and STING expression levels are positively correlated with the efficacy of NACI in HNSCC, playing crucial roles in immune activation. These findings highlight potential strategies that could guide the development of personalized precision medicine for NACI in HNSCC.

## Data Availability

Publicly available datasets were analyzed in this study. This data can be found here: https://www.ncbi.nlm.nih.gov/geo/query/acc.cgi?acc=GSE229289.
