# Supplementary material for: High cGAS-STING expression associates with improved efficacy of neoadjuvant chemo-immunotherapy in head and neck squamous cell carcinoma
Source: Front Oncol. 2025 Jul 15;15:1584061. doi: 10.3389/fonc.2025.1584061 (PMC12303820; doi:10.3389/fonc.2025.1584061)
Supplement: Supplementary file 1 [file Table1.docx]

**Table S1**. Correlation between clinical characteristics and cGAS-STING expression

|  | cGAS | | | STING | | |
| --- | --- | --- | --- | --- | --- | --- |
|  | High | Low | p value | High | Low | p value |
| **Age** |  |  | 0.74 |  |  | 0.74 |
| ≤60 | 12 | 11 |  | 12 | 11 |  |
| >60 | 7 | 8 |  | 7 | 8 |  |
| **Gender** |  |  | ＞0.99 |  |  | ＞0.99 |
| Male | 18 | 19 |  | 18 | 19 |  |
| Female | 0 | 1 |  | 0 | 1 |  |
| **Alcohol consumption** |  |  | 0.60 |  |  | 0.60 |
| No | 1 | 3 |  | 1 | 3 |  |
| Yes | 18 | 16 |  | 18 | 16 |  |
| **Smoking history** |  |  | ＞0.99 |  |  | ＞0.99 |
| No | 4 | 4 |  | 4 | 4 |  |
| Yes | 15 | 15 |  | 15 | 15 |  |
| **Tumor site** |  |  | 0.08 |  |  | 0.46 |
| Nasal Cavity and Sinus | 2 | 0 |  | 2 | 0 |  |
| Oropharynx | 5 | 4 |  | 5 | 4 |  |
| Hypopharynx | 6 | 13 |  | 8 | 11 |  |
| Larynx | 6 | 2 |  | 4 | 4 |  |
| **Histological classification** |  |  | 0.17 |  |  | 0.69 |
| Poorly | 7 | 10 |  | 7 | 10 |  |
| Moderately | 6 | 6 |  | 6 | 6 |  |
| Well | 3 | 0 |  | 2 | 1 |  |
| **HPV status** |  |  | 0.09 |  |  | 0.19 |
| negative | 13 | 11 |  | 12 | 12 |  |
| positive | 1 | 6 |  | 1 | 6 |  |
| **T stage** |  |  | 0.18 |  |  | 0.18 |
| 1-2 | 5 | 10 |  | 5 | 10 |  |
| 3-4 | 14 | 9 |  | 14 | 9 |  |
| **N stage** |  |  | ＞0.99 |  |  | ＞0.99 |
| 0 | 5 | 4 |  | 5 | 4 |  |
| 1-2 | 14 | 15 |  | 14 | 15 |  |
| **Clinical stage** |  |  | ＞0.99 |  |  | ＞0.99 |
| I-II | 2 | 3 |  | 2 | 3 |  |
| III-IV | 17 | 16 |  | 17 | 16 |  |

**Table S2**. Correlation between clinical characteristics and NACI efficacy in HNSCC

|  | Radiographic response | | | Pathological response | | |
| --- | --- | --- | --- | --- | --- | --- |
|  | R | NR | p value | MPR | IPR | p value |
| **Age** |  |  | 0.74 |  |  | 0.14 |
| ≤60 | 11 | 12 |  | 16 | 4 |  |
| >60 | 8 | 7 |  | 6 | 5 |  |
| **Gender** |  |  | ＞0.99 |  |  | ＞0.99 |
| Male | 18 | 19 |  | 21 | 9 |  |
| Female | 1 | 0 |  | 1 | 0 |  |
| **Alcohol consumption** |  |  | 0.60 |  |  | 0.19 |
| No | 1 | 3 |  | 1 | 2 |  |
| Yes | 18 | 16 |  | 21 | 7 |  |
| **Smoking history** |  |  | ＞0.99 |  |  | ＞0.99 |
| No | 4 | 4 |  | 4 | 2 |  |
| Yes | 15 | 15 |  | 18 | 7 |  |
| **Tumor site** |  |  | 0.67 |  |  | 0.11 |
| Nasal Cavity and Sinus | 1 | 1 |  | 2 | 0 |  |
| Oropharynx | 6 | 3 |  | 8 | 0 |  |
| Hypopharynx | 9 | 10 |  | 8 | 6 |  |
| Larynx | 3 | 5 |  | 4 | 3 |  |
| **Histological classification** |  |  | 0.69 |  |  | 0.61 |
| Poorly | 8 | 9 |  | 10 | 5 |  |
| Moderately | 7 | 5 |  | 6 | 3 |  |
| Well | 1 | 2 |  | 2 | 0 |  |
| **HPV status** |  |  | ＞0.99 |  |  | 0.28 |
| negative | 10 | 14 |  | 13 | 7 |  |
| positive | 3 | 4 |  | 4 | 0 |  |
| **T stage** |  |  | 0.50 |  |  | 0.70 |
| 1-2 | 6 | 9 |  | 8 | 4 |  |
| 3-4 | 13 | 10 |  | 14 | 5 |  |
| **N stage** |  |  | ＞0.99 |  |  | 0.64 |
| 0 | 4 | 5 |  | 5 | 1 |  |
| 1-2 | 15 | 14 |  | 17 | 8 |  |
| **Clinical stage** |  |  | ＞0.99 |  |  | ＞0.99 |
| I-II | 3 | 2 |  | 3 | 1 |  |
| III-IV | 16 | 17 |  | 19 | 8 |  |
